# Preparation and Efficacy of a Live Newcastle Disease Virus Vaccine Encapsulated in Chitosan Nanoparticles

**DOI:** 10.1371/journal.pone.0053314

**Published:** 2012-12-28

**Authors:** Kai Zhao, Gang Chen, Xing-ming Shi, Ting-ting Gao, Wei Li, Yan Zhao, Feng-qiang Zhang, Jin Wu, Xianlan Cui, Yun-Feng Wang

**Affiliations:** 1 Laboratory of Microbiology, College of Life Science, Heilongjiang University, Harbin, China; 2 Division of Avian Infectious Diseases, State Key Laboratory of Veterinary Biotechnology, Harbin Veterinary Research Institute, CAAS, Harbin, China; 3 Animal Health Laboratory, Department of Primary Industries, Parks, Water and Environment, Tasmania, Australia; Virginia Polytechnic Institute and State University, United States of America

## Abstract

**Background:**

Newcastle disease (ND) is a highly contagious viral disease of poultry caused by pathogenic strains of the Newcastle disease virus (NDV). Live NDV vaccines are administered by drinking water, eyedrops or coarse aerosol spray. To further enhance mucosal immune responses, chitosan nanoparticles were developed for the mucosal delivery of a live NDV vaccine.

**Methodology/Principal Findings:**

A lentogenic live-virus vaccine (strain LaSota) against NDV encapsulated in chitosan nanoparticles were developed using an ionic crosslinking method. Chitosan nanoparticles containing the lentogenic live-virus vaccine against NDV (NDV-CS-NPs) were produced with good morphology, high stability, a mean diameter of 371.1 nm, an encapsulation rate of 77% and a zeta potential of +2.84 mV. The Western blotting analysis showed that NDV structural proteins were detected in NDV-CS-NPs. The virus release assay results of NDV-CS-NPs indicated that NDV was released from NDV-CS-NPs. Chickens immunized orally or intranasally with NDV-CS-NPs were fully protected whereas one out of five chickens immunized with the LaSota live NDV vaccine and three out of five chickens immunized with the inactivated NDV vaccine were dead after challenge with the highly virulent NDV strain F48E9.

**Conclusions/Significance:**

NDV-CS-NPs induced better protection of immunized specific pathogen free chickens compared to the live NDV vaccine strain LaSota and the inactivated NDV vaccine. This study lays a foundation for the further development of mucosal vaccines and drugs encapsulated in chitosan nanoparticles.

## Introduction

Newcastle disease (ND) is a highly contagious viral disease of poultry that is characterized by respiratory, nervous, enteric, and reproductive infections. The causative agent of the infectious disease is the virulent ND virus (vNDV), which belongs to the genus *Avulavirus* within the family *Paramyxoviridae* [1]. ND has been a devastating disease and it remains one of the major problems in existing and developing poultry industries in many countries [2]. There are no treatments available for ND; however, vaccination is an effective method of control.

Current vaccination programs for ND include the use of lentogenic live-virus vaccines or inactivated vaccines to induce protective immunity while producing minimal adverse effects in chickens [3]. Inactivated oil-emulsion vaccines have poor immunogenicity and elicit a lower antibody response in the mucosal secretion by the single immunization. Therefore, inactivated oil-emulsion vaccines need to be administered by multiple immunizations to obtain a lasting protective immune response. Live-virus vaccines for ND may be administered by drinking water, aerosol or eyedrops. Many infections occur on or through mucosal surfaces; therefore, mucosal immunity is often the key to controlling initial infections with such pathogens. The most efficient mucosal immune responses are generated when vaccines are administered mucosally, although the majority of vaccines are administered parenterally [4]. However, this type of vaccines must be used at a high dosage with adjuvants, which increases the cost of vaccination.

To meet the need for effective and low-cost vaccine delivery systems for mass vaccination, a number of new techniques have recently been developed to introduce the virus into cells. One of the approaches is the non-viral delivery system. Cationic lipids and cationic polymer have been employed as non-viral gene transfer agents. In pigs and mice, DNA vaccines have been delivered orally using a variety of carriers [5]. Chitosan, the main derivative of chitin, is a linear polymer that comprises repeating units of β-(1–4)-2-amino-2-deoxy-D-glucopyranose units [6], and it was proved to be non-toxic in experimental animals [7] and humans [8]. As a polyatomic, nontoxic, biodegradable, and biocompatible polymer, chitosan has attracted significant attention and can encapsulate a range of bioactive agents including proteins and peptides.

Current research on chitosan focuses on its usage as a novel delivery vehicle, and scaffold for targeted delivery and tissue engineering applications [9], [10]. The amino and carboxyl groups in the chitosan molecule interact with glycoprotein in mucus to form a hydrogen bond, which produces an adhesive effect. As mucoprotein in mucus is positively charged, chitosan and mucus are attracted to each other to prolong the *in vivo* retention and release time of drugs and to improve drug bioavailability [11].

According to the principle of ionic crosslinking, nanoparticles can be formed by intra and inter molecular crosslinking between the positively charged chitosan and the negatively charged sodium tripolyphosphate (TPP). A novel mucosal delivery system based on chitosan nanoparticles was used in this study. These nanoparticles may act as mediators of protein antigen or plasmid DNA, and they may protect against biological degradation by nucleases [12]–[14]. Recently, chitosan nanoparticles have been utilized to sustain the release of various drugs, including oligonucleotides [15]–[19].

Chitosan nanoparticles can be prepared using various formulation methods to release an active ingredient (such as proteins, peptides and DNA vaccines) in a sustained manner over a prolonged period. The ionic crosslinking method has received significant attention in recent years due to the preparation of chitosan nanoparticles containing proteins, peptides and vaccines because the processes used are simple and mild for proteins and viruses. They do not use chemical cross linkers and they avoid using organic solvents and high temperatures [20].

In this study, NDV encapsulated chitosan nanoparticles were prepared using an ionic crosslinking method to enhance the efficacy of a lentogenic live-virus vaccine against ND. The immune response elicited in specific pathogen free (SPF) chickens immunized with chitosan nanoparticles containing a lentogenic live-virus vaccine (strain LaSota) against ND was evaluated. The safety of the chitosan nanoparticles was tested by *in vitro* cell cytotoxicity assay and *in vivo* safety tests in chickens. This work lays a foundation for future work on a range of mucosal delivery systems including those for vaccines and drugs.

## Materials and Methods

### Ethics Statement

Care of laboratory animals and animal experimentation were conducted in accordance with animal ethics guidelines and approved protocols. All animal studies were approved by the Animal Ethics Committee of Harbin Veterinary Research Institute of the Chinese Academy of Agricultural Sciences (CAAS) and the Animal Ethics Committee of Heilongjiang Province (SYXK (H) 2006-032).

### Materials

NDV vaccine strain LaSota and 10-day-old SPF embryos were provided by Harbin Pharmaceutical Group Bio-vaccine Co. Ltd. Seven-day-old SPF chickens were provided and raised by Harbin Pharmaceutical Group Bio-vaccine Co. Ltd. Commercial NDV strain LaSota live-virus vaccine (L/N: 200805) and inactivated oil emulsion vaccine against ND (L/N: 200805) were purchased from Harbin Pharmaceutical Group Bio-vaccine Co. Ltd. NDV F48E9 strain was provided by State Key Laboratory of Veterinary Biotechnology, Harbin Veterinary Research Institute, CAAS.

Chitosan (with a molecular weight of 71.3 kDa and deacetylation degree of 80%), MTT, RPMI 1640 medium and ConA were purchased from Sigma Ltd. (St. Louis, MO, USA). Sodium tripolyphosphate (TPP) was purchased from Tianjin Institute of Guangfu Refined Chemicals (Tianjin, China), Agarose and SDS from GIBCOBRL Ltd (New Delhi, India), Cell Counting Kit-8 (CCK-8) from Dojindo (Tokyo, Japan), and NDV IgA ELISA Kit from Rapidbio Co. Ltd. (Montgomery, TX, USA).

### Purification of NDV

Briefly, the NDV vaccine strain LaSota was propagated in the allantoic cavity of embryonated SPF eggs at 37°C for 120 h post infection. The allantoic fluid was harvested and centrifuged at 3,000 g/min, 5,000 g/min, and 8,000 g/min for 30 min at 4°C, respectively. The upper liquid phase was collected and concentrated at 30,000 g/min for 2.5 h at 4°C. The purified virus was resuspended in phosphate-buffered saline (PBS; pH 7.4) and stored at −20°C until required.

### Preparation of Chitosan Solutions and TPP Solutions

According to the principle of ionic crosslinking, nanoparticles can be formed by intra and inter molecular crosslinking between positively charged chitosan and negatively charged TPP. In this study, we used ionic crosslinking method to develop a novel mucosal delivery system based on chitosan nanoparticles. Chitosan solution was prepared by slowly dissolving chitosan with sonication in an aqueous solution of 4% acetic acid until the solution was transparent. Once dissolved, the chitosan solution was diluted with deionized water and filtered through a 0.22 µm filter to produce a final chitosan stock solution of 0.4% (w/v, 4 mg/ml) with a viscosity of 2.54±0.1 centi Poise (cP) as measured using a Model DV-III Plus Programmable Rheometer (Brookfield Engineering Laboratories, Middleboro, MA, USA). The chitosan solution stock was diluted with deionized water to produce chitosan solutions of different concentrations at 0.5 mg/ml, 1.0 mg/ml, and 2.0 mg/ml. TPP was dissolved in deionized water at the concentrations of 0.5 mg/ml, 1.0 mg/ml, and 1.5 mg/ml.

### Preparation of Chitosan-NDV Nanoparticles

The chitosan-NDV nanoparticles were prepared using an ionic cross linking method. Two and a half milliliters of NDV solution (with 1.0% sucrose, m/v) were added drop by drop to 5 ml of chitosan solution under magnetic stirring. Subsequently, 2.5 ml of TPP solution was added to the above solution under magnetic stirring at room temperature. The chitosan-NDV nanoparticles were separated by centrifugation at 10,000 g/min for 30 min at 4°C and the supernatant was discarded. The chitosan-NDV nanoparticles were washed with distilled water. After centrifugation the nanoparticles were freeze dried for 24 h using a vacuum freeze-drying machine (BOC Edwards Co. Ltd, UK) and stored at −20°C until required for further analysis. These nanoparticles were named NDV-CS-NPs.

### Optimization of the NDV-CS-NPs Preparation Conditions

Several factors that affected the characteristics of the NDV-CS-NPs including the concentrations of chitosan and TPP solutions, NDV/CS ratio (v/v), the agitating velocity and time were tested. Single factor experiments on the effects of preparation condition of NDV-CS-NPs were conducted. Based on the results obtained in single factor experiments, the key factors were NDV/CS ratio (v/v), TPP concentration, and chitosan concentration. The agitating velocity was 1,000 g/min, and agitating time was 10 min. The orthogonal experiments with three factors and three levels were designed. All the key factors were examined, and morphology, size and zeta potential were used as indicators. The orthogonal experimental scheme is shown in Table 1.

**Table 1:** Optimization of NDV-CS-NPs preparation conditions.

Experiment No.	A Chitosan density(mg/ml)	B TPP concentration(mg/ml)	C NDV/CS ratio](ml/ml)	Encapsulationefficiency (%)
1	0.5	0.5	1∶4	51%
2	0.5	1.0	1∶2	49%
3	0.5	2.0	1∶1	53%
4	1.0	0.5	1∶2	77%
5	1.0	1.0	1∶1	60%
6	1.0	2.0	1∶4	59%
7	1.5	0.5	1∶1	67%
8	1.5	1.0	1∶4	72%
9	1.5	2.0	1∶2	64%
K_1_	57.48%	65.53%	54.94%	
K_2_	65.3%	60.3%	63.3%	
K_3_	64.7%	58.7%	60.7%	
R	7.82%	6.83%	8.36%	

Note: K_1_, K_2_ and K_3_ represent the mean value of factors at 1, 2 and 3 levels, respectively. R represents range.

### Morphology, Size and Zeta Potential Measurement of NDV-CS-NPs

A JEM-1200EX transmission electron microscopy (TEM) (Hitachi Ltd, Japan) was used to observe nanoparticle morphology. The colloidal suspension of chitosan nanoparticles was sonicated for 2 min for better dispersion and to prevent particle agglomeration on the copper grid. One drop of colloidal suspension was spread onto a carbon-coated copper grid, which was then dried at room temperature for TEM analysis. The particle sizes and zeta potentials of the NDV-CS-NPs were measured using a Zeta Sizer 2000 from Malvern Instruments (Southborough, MA, USA). Samples were diluted with deionized water, and the measurements were conducted at a scattering angle of 90 degrees and a temperature of 25°C. The diameter was calculated from the autocorrelation function of the intensity of the light scattered from the particles, assuming that the particles had a spherical form.

### Evaluation of Encapsulation Efficiency

The encapsulation efficiency of NDV-CS-NPs was determined by separating the nanoparticles from the aqueous medium containing the free virus using centrifugation at 10,000 g/min for 30 min at 4°C. The amount of free NDV in the supernatant was measured spectrophotometrically by a standard Bradford assay using Coomassie Brilliant Blue G-250 with a spectrophotometer at 595 nm. The encapsulation efficiency (EE) of the nanoparticles was calculated as [21]:
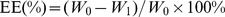
Where *W*_0_ is total amount of NDV added, *W*_1_ is amount of free NDV. All the measurements were performed in triplicate.

### Determination of Viral Titer in the NDV-CS-NPs

The viral titer was calculated by measuring 50% embryo infectious dose (EID_50_). One hundred micrograms of dried NDV-CS-NPs were added into 10 ml of the PBS buffer (pH 7.2). Subsequently, 2.0 ml of trypsin was added to the above solution and then digested for 72 h at 4°C with a concentration of trypsin solution at 0.25% and centrifuged for 5 min at 1,200 g/min. The obtained supernatant was diluted with sterile saline by a serial of 10-fold dilution, and 10^−6^, 10^−7^ and 10^−8^ were inoculated into the allantoic cavity of five 10-day-old SPF chicken embryonated eggs respectively and incubated at 37°C for 120 h. Dead embryos were discarded at 24 h post inoculation. Eggs were chilled at 4°C and the allantoic fluid was harvested and tested for haemagglutination (HA). The results were considered positive when HA titers were equal to or greater than 7 log2. The EID_50_ was calculated according to the Reed–Muench method. Five 10-day-old SPF chicken embryonated eggs that were inoculated with trypsin solution served as a control group.

### Analysis of NDV Structural Proteins in the NDV-CS-NPs

One hundred micrograms of dried NDV-CS-NPs were added into 1 ml of PBS buffer (pH 7.2) and vortexed for 30 min to ensure that the NDV was fully release. After centrifugation for 10 min at 10,000 g/min, the supernatant containing NDV was collected and stored at −20°C. The NDV structural proteins in the NDV-CS-NPs and the purified NDV were compared for equivalent concentration before and after encapsulation, by Western blotting. Briefly, the supernatant was mixed with 2×SDS buffer and boiled for 5 min. After cooling to room temperature, 20 µl of the samples were loaded onto a 12% SDS-PAGE gel. After electrophoresis, the proteins were transferred to a nitrocellulose membrane using a Bio-Rad semi-dry unit. The membrane was washed with PBS and blocked with 5% skim milk in PBS overnight, and then incubated with NDV-positive chicken serum (HI antibody titer 8.0 log2) at a 1∶10 dilution for 1 h. After washing with PBS, HRP-labeled goat-anti-chicken IgY antibody was added at a dilution of 1∶10000 for 1 h.

### *In vitro* and *in vivo* Virus Release and Replication of Chitosan Encapsulated NDV

The release of NDV from NDV-CS-NPs was studied using the Coomassie Brilliant Blue G-250 method. The mixture of 0.1 g of dried NDV-CS-NPs and 2 ml of PBS buffer (pH 7.2) was stirred, followed by incubation at 37°C for 100 g/min. Samples were withdrawn at regular time intervals, centrifuged at 10,000 g/min for 30 min at 4°C, and then measured with the spectrophotometer at 595 nm. After the sample was taken each time, the same volume of fresh PBS was added. The experiments were performed in triplicate. An NDV release curve of NDV-CS-NPs was plotted against the release time at the X-axis and the accumulative release amount at the Y-axis [22].

Eighty 30-day-old SPF chickens were randomly divided into four groups. Chickens in Group 1 were inoculated with 50 µl of the lentogenic live-virus NDV vaccine strain LaSota (10^6.5^ EID_50_/0.1 ml) by nasal drops. Chickens in Group 2 were inoculated with 50 µl of NDV-CS-NPs (10^6.5^ EID_50_/0.1 ml) by nasal drops. Chickens in Group 3 were orally treated with 50 µl of NDV-CS-NPs. Chickens in Group 4 were orally treated with 50 µl of PBS.

Samples of the kidneys and Harderian glands of two chickens were randomly taken in each group at 3, 5, 7, 12 and 18 days post immunization and kept at −70°C. Meanwhile, cloacal swab samples were taken at 1, 3, 5, 7, 10, 13, and 16 days post immunization and kept at −20°C for detection of the virus shedding.

Samples of kidneys and Harderian glands with the same weight were ground and the samples of the cloacal swabs were frozen and thawed three times and then centrifuged at 1200 g/min for 5 min. The supernatant was diluted with sterile saline in a 10×dilution series (10^−2^, 10^−3^, 10^−4^, 10^−5^, and 10^−6^) for inoculation in the allantoic cavity of 15-day-old embryonated eggs (five eggs per dilution). The inoculated embryonated eggs were observed for the lesions and embryo deaths, and the EID_50_ was calculated according to the Reed-Muench method.

### *In vitro* Cytotoxicity of NDV-CS-NPs

Chicken embryo kidney (CEK) cells were prepared from the kidneys of 18-day-old chicken embryos and cultured in DMEM, then diluted to 1×10^6^ cells/ml [23]. Cells were transferred to 96-well plates at 100 µl per well and cultured at 37°C for 5 h. After 100 µl of NDV-CS-NPs (diluted in DMEM culture at 1.5 µg/ml) were added into the wells, the plates were incubated at 37°C for 48 h. Blank chitosan nanoparticles were used as a negative control and original NDV fluid was used as a positive control. Ten microliters of WST-8 reagent was added and incubated for 4 h. The optical density at 570 nm (OD570) was measured to determine survival rate of the cells, which was calculated as [24]:
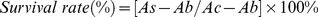
Where As represents the test wells (containing the cell medium, WST-8 and NDV-CS-NPs); Ac represents the control wells (containing blank chitosan nanoparticles or NDV original fluid); and Ab represents the blank wells (containing the cell medium only). WST-8 is reduced by dehydrogenases in cells to obtain a yellow colored product (formazan), which can be directly used for cytotoxicity assay.

### Safety Test of NDV-CS-NPs in SPF Chickens

Thirty 4-week-old SPF chickens (Harbin Pharmaceutical Group Bio-vaccine Co. Ltd, Harbin, China) were randomly separated into two groups. Chickens in Group 1 were immunized orally with 0.25 ml of NDV-CS-NPs. Chickens in Group 2 were immunized by eyedrops with 0.25 ml of the ND LaSota lentogenic live-virus vaccine (Harbin Pharmaceutical Group Bio-vaccine Co. Ltd, Harbin, China). Any abnormal changes in the chickens were observed and recorded for three weeks.

### Immunization of SPF Chickens

Ninety 30-day-old SPF chickens were randomly divided into six groups. Chickens in Group 1 were immunized intranasally with 50 µl of ND LaSota strain lentogenic live-virus vaccine (10^6.0^ EID_50_/ml). Chickens in Group 2 were immunized intramuscularly with 50 µl of inactivated NDV vaccine (10^10^ EID_50/_ml). Chickens in Group 3 were immunized intranasally with 60 µl of NDV-CS-NPs. Chickens in Group 4 were immunized orally with 60 µl of NDV-CS-NPs. Chickens in Group 5 were immunized orally with 50 µl of blank CS-NPs. Chickens in Group 6 were treated with physiological saline. Blood was collected from the wing vein at 1, 2, 3, 4, 5, 6, 7, 8, 10 and 12 weeks post immunization.

### Lymphocyte Proliferation Test

Lymphocyte proliferation of the immunized chickens was conducted using MTT (3-(4, 5)-dimethylthiahiazo (-z-y1)-3, 5-diphenytetrazoliumromide) colorimetric assay as previously described [25]. The spleens of the immunized chickens were removed aseptically at 6 and 8 weeks post the immunization. The spleens were filtered through 200 micron copper mesh, and single cell suspensions were prepared from the filtrate. Erythrocytes were lysed using 0.75% Tris-NH_4_Cl (pH 7.4). Spleen cells were suspended in RPMI 1640 medium containing 10% fetal serum and then diluted to 4×10^6^ cells^/^ml. Cell suspensions were transferred to 96-well plates at 100 µl per well. Twenty microliters of purified and inactivated NDV were used as a specific stimulating antigen. Wells containing 20 µg/ml of ConA were used as positive controls, and those without stimulating antigen were used as negative controls. All of the cells were cultured at 5% CO_2_ and 37°C for 44 h, and then 20 µl of MTT was added into each well, followed by culturing for another 5 h. One hundred microliters of DMSO were added into each well. All experiments were repeated three times and each was measured in triplicate. The OD_570_ was measured to determine the stimulation index using the following formula:

Stimulation index = average OD value in the test group divided by average OD value in the control group.

### HI Antibodies in Serum

Half a milliliter of blood samples was centrifuged at 2,200 g/min for 10 min at 4°C to collect serum. Each serum sample was transferred to 1.5 ml of prelabeled eppendorf tube and preserved at 4°C until tested individually by hemagglutination inhibition test.

### IgA Antibody in Intestinal Mucus

To evaluate the mucosal immune response, intestinal mucus was collected from two euthanized chickens once a week. To collect the intestinal mucus, 4 ml of PBS was gently instilled into the duodenum lumen, allowing it to pass through the length of the duodenum before being collected in prelabeled tubes [26]. The ND IgA antibody in the intestinal mucus was detected by an NDV IgA ELISA Kit from Rapidbio Co. Ltd (West Hills, CA, USA).

### Protective Efficacy

An experiment was conducted to evaluate the protective efficacy after inoculation with different vaccines. When the level of ND serum antibodies in every immune group increased to 6.0 log2, five chickens were selected at random from the six groups and infected intramuscularly with the highly virulent NDV strain F48E9 for challenge studies with a viral titer of 10^8^ EID_50_/0.1 ml. Clinical signs of disease and mortality were monitored on a daily basis for seven days.

### Statistical Analysis

All experiments were repeated three times and each measured in triplicate. Data were presented as mean values ± standard deviation (SD). Mean values were analyzed using the one-sided Student’s *t*-test. Differences were considered to be statistically significant at *p*<0.05.

## Results

### Preparation and Physical and Immunological Properties of NDV-CS-NPs

The factors that affected the NDV-CS-NP preparation were ranked from high to low impact: NDV/CS ratio>TPP concentration>chitosan concentration. The encapsulation efficiency ranged from 49% to 77%, depending on the preparation conditions. The optimal combination for NDV-CS-NPs was a chitosan concentration of 1.0 mg/ml, a TPP concentration of 0.5 mg/ml and a NDV/CS ratio of 1∶2 (Table 1). A validation test was performed in which chitosan nanoparticles containing the lentogenic ND live-virus vaccine were prepared according to the above optimal combination. The viral titer was 10^6.5^ EID_50_/0.1 ml in NDV-CS-NPs (Table 2).

**Table 2:** Determination of NDV titer in the NDV-CS-NPs.

Ratio ofvirus dilution	Observational result			Accumulative result		
	Cytopathiceffect	No cytopathiceffect	Cytopathiceffect (%)	Cytopathiceffect	No cytopathiceffect	Cytopathiceffect (%)
10^−6^	4	1	80	5	1	83.3
10^−7^	1	4	20	1	5	16.7
10^−8^	0	5	0	0	10	0

The viral titer in the NDV-CS-NPs was 10^6.5^ EID_50_/0.1 mL as calculated using the Reed-Muench method.

**Figure 1:**
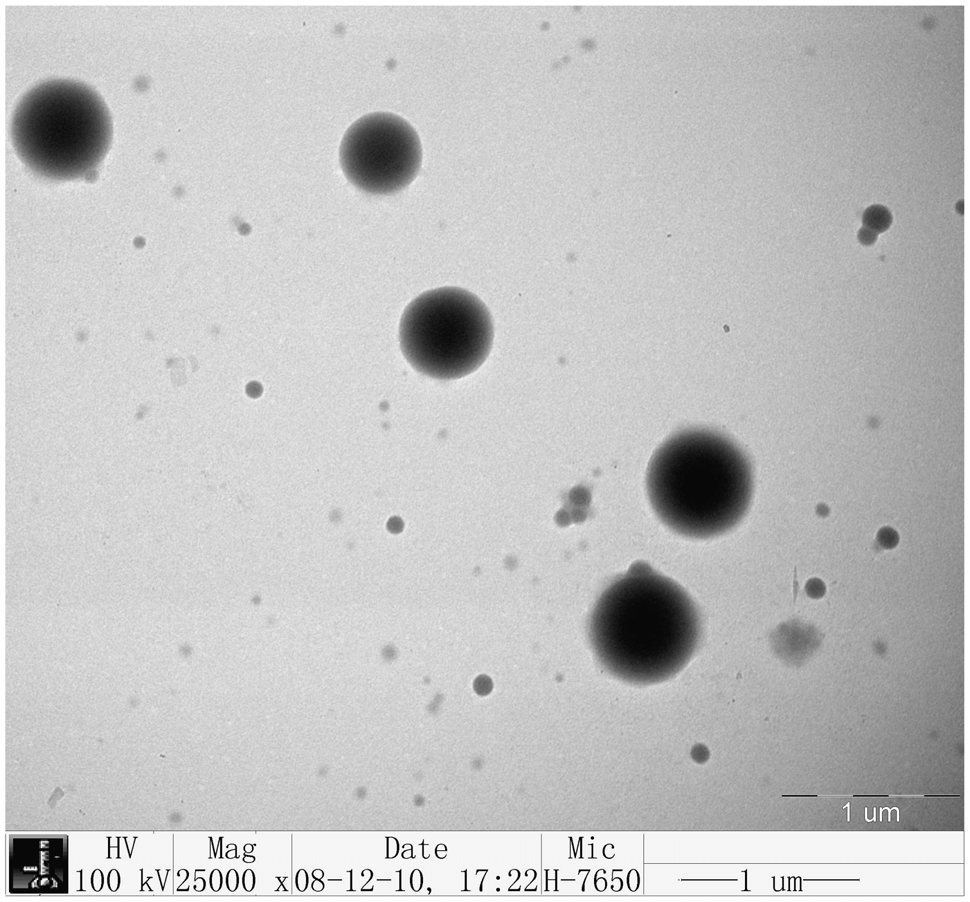
Transmission electron microscopy photomicrograph of the chitosan-NDV nanoparticles prepared by an ionic cross linking method under the optimized conditions. The TEM photomicrograph (magnification 25,000×) showed that the prepared nanoparticles had a regular round shape and good dispersion, but did not have adhesion or subsidence damage. The sizes of particles were between 200 nm and 500 nm.

Typical NDV-CS-NPs showed a spherical and polydisperse nature as revealed by the TEM (Figure 1). The morphology of the prepared nanoparticles had a regular round shape and good dispersion, but it did not have adhesion or subsidence damage. The measurement of these particles showed a fairly even distribution from 147.72 to 594.4 nm with an average particle size of 371.1 nm, and a zeta potential of +2.84 mV.

**Figure 2:**
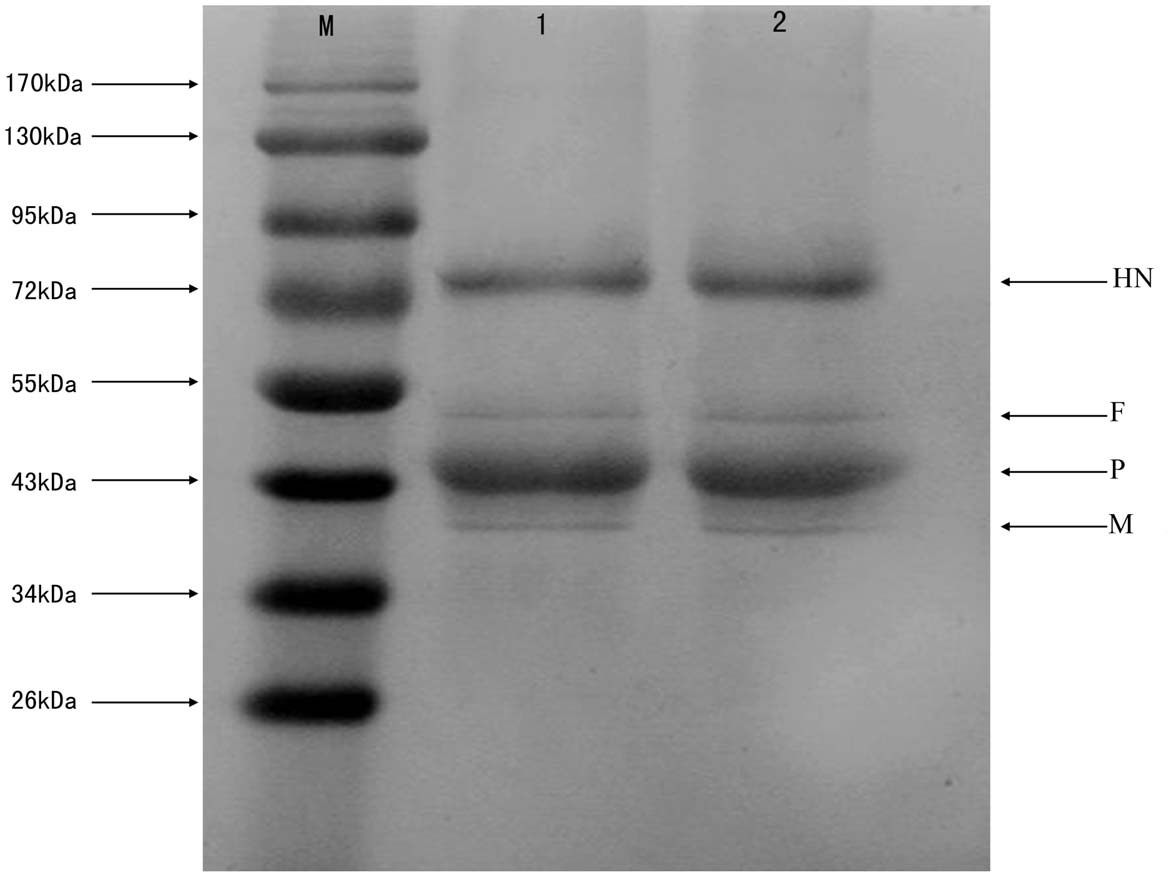
Detection of the NDV structural proteins after encapsulation by Western blot. M: Protein marker; 1: Original NDV fluid; 2: NDV recovered from the NDV-CS-NPs. Four positive reaction bands were detected at 75 kDa (hemagglutinin-neuraminidase protein, HN), 54 kDa (fusion protein, F), 45 kDa (phosphoprotein, P), and 41 kDa (matrix protein, M).

Western blotting analysis was performed to detect the NDV structural proteins after encapsulation. Four positive reaction bands similar in size to NDV structural proteins were detected at 75 kDa (hemagglutinin-neuraminidase protein, HN), 54 kDa (fusion protein, F), 45 kDa (phosphoprotein, P), and 41 kDa (matrix protein, M) in NDV-CS-NPs before and after encapsulation (Figure 2). These results indicated that there was no damage to NDV proteins after encapsulation.

### *In vitro* and *in vivo* Virus Release and Replication of NDV-CS-NPs

**Figure 3:**
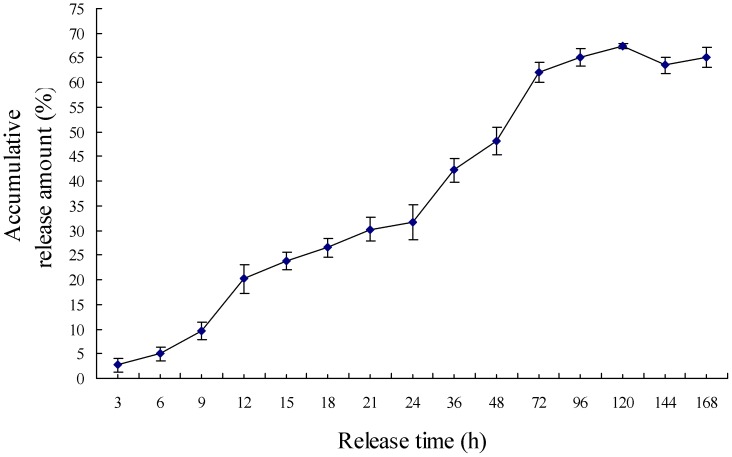
*In vitro* release profiles of NDV from NDV-CS-NPs in PBS (pH 7.2). The experiment was repeated three times and each measured in triplicate. Mean values were analyzed using the Student’s test. Data were presented as mean values ± SD.

The release amount of NDV from NDV-CS-NPs increased quickly from 0 h to 72 h, but it did not change significantly after 72 h. This indicated that the burst release mainly occurred during the first 72 h, and the process after 72 h was mainly an accumulative release of NDV from NDV-CS-NPs (Figure 3).

NDV was detected by virus isolation in embryonated eggs on day 3 in Group NDV-CS-NPs and Group LaSota vaccine, and it peaked at week 1 post immunization. Virus replication was still detected in the kidney and Harderian gland of immunized SPF chickens 18 days post immunization (Table 3) whereas virus shedding was detected in the cloaca of immunized SPF chickens until 13 days post immunization (Table 4). There was no significant difference in the amount of virus in tissue samples and in the cloacal swabs (*p*>0.05) of chickens immunized with NDV-CS-NPs orally or by nasal drops compared with Group LaSota vaccine by nasal drops (Table 3 and Table 4).

**Table 3:** Detection of virus replication in the tissues and organs of immunized SPF chickens.

Groups*	Tissues	Days post immunization				
		3	5	7	12	18
LaSota livevaccine intranasal^a^	Kidney	10^2.4^	10^3.4^	10^5.3^	10^4.5^	10^2.7^
	Harderian gland	10^2.4^	10^3.3^	10^5.8^	10^4.3^	10^2.4^
NDV-CS-NPs intranasal^a^	Kidney	10^2.3^	10^3.8^	10^5.2^	10^4.3^	10^2.4^
	Harderian gland	10^2.4^	10^3.8^	10^5.3^	10^4.8^	10^2.4^
NDV-CS-NPs oral^a^	Kidney	10^2.2^	10^3.4^	10^5.1^	10^4.6^	10^2.9^
	Harderian	10^2.2^	10^3.6^	10^5.2^	10^4.9^	10^2.6^
Control^b^	Kidney	0	0	0	0	0
	Harderian	0	0	0	0	0

* Each group with same lower case superscript letters shows no significant difference (*p*>0.05). Amount of NDV detected in in the tissues and organs is expressed as EID_50_/0.1 ml.

**Table 4:** Detection of NDV shedding in cloacal swabs of immunized SPF chickens.

Groups*	Days post immunization						
	1	3	5	7	10	13	16
LaSota live vaccine intranasal^a^	0	10^2.4^	10^3.6^	10^4.8^	10^3.8^	10^3.7^	0
NDV-CS-NPs intranasal^a^	0	10^2.0^	10^3.7^	10^4.4^	10^4.2^	10^3.3^	0
NDV-CS-NPs oral^a^	0	10^1.8^	10^3.6^	10^4.3^	10^4.2^	10^3.6^	0
Control^b^	0	0	0	0	0	0	0

* Each group with the same lower case superscript letters shows no significant difference (*p*>0.05). The amount of NDV detected in cloacal swabs is expressed as EID_50_/0.1 ml.

### Evaluation of the Safety of NDV-CS-NPs

Cytotoxicological analysis is an important index for evaluating the safety of a drug delivery system. The survival rate was 91.5±5.3%, and no significant changes in cell morphology were observed compared to the control cells. The result showed little cytotoxicity by NDV-CS-NPs and the safety level was high.

No nervous signs, clinical symptoms or necropsy lesions were observed in chickens immunized with 0.25 ml of either NDV-CS-NPs or the live lentogenic NDV LaSota vaccine within 3 weeks post inoculation, revealing the high level of safety of NDV-CS-NPs in chickens.

### Lymphocyte Proliferation Test

The cell-mediated immune responses of immunized chickens were assessed using the stimulating index (SI) in the lymphocyte proliferation test at week 4 and 8 post immunization (Table 5). Five chickens from each group were selected. The stimulation indices of chickens immunized orally or intranasally with NDV-CS-NPs were significantly higher than those of chickens immunized intramuscularly with the inactivated NDV vaccine, intranasally with the LaSota live vaccine, or orally with blank CS-NPs (*p*<0.01), respectively. However, no significant differences were found between chickens immunized orally or intranasally with NDV-CS-NPs (*p*<0.05). Chickens immunized intramuscularly with the inactivated NDV vaccine or intranasally with the LaSota live vaccine had significantly higher stimulation indices compared with chickens immunized with blank nanoparticles (*p*<0.05). However, no significant differences were found between chickens immunized intramuscularly with the inactivated NDV vaccine and chickens immunized intranasally with the LaSota live vaccine (*p*>0.05) (Table 5). These findings indicated that NDV-CS-NPs induced the best T cell immune response in immunized chickens.

**Table 5:** Stimulation indices of T lymphocyte proliferation in SPF chickens immunized with NDV-CS-NPs, the LaSota live vaccine, or the inactivated NDV vaccine.

Groups	Weeks post immunization	
	4	8
NDV-CS-NPs oral	4.489±0.165^a^	2.904±0.107^a^
NDV-CS-NPs intranasal	3.876±0.187^a^	2.619±0.076^a^
Inactivated NDV vaccine intramuscular	2.182±0.184^b^	2.173±0.080^b^
LaSota live vaccineintranasal	1.794±0.076^b^	1.888±0.059^b^
Blank CS-NPs oral	1.129±0.115^c^	0.364±0.055^c^

Values are presented as mean ± S.D. of five experiments in each group. Values within the same column with the same lower case letter in the superscript are not significantly different (*p*>0.05; Student’s t-test).

The stimulating indices of NDV-CS-NPs oral group and NDV-CS-NPs intranasal group were significantly higher than those of inactivated NDV vaccine intramuscular group, LaSota live vaccine intranasal group and blank CS-NPs oral group (*p*<0.01) whereas the stimulating indices of inactivated NDV vaccine intramuscular group and LaSota live vaccine intranasal group were significantly higher than that of the CS-NPs oral group (*p*<0.05) at weeks 4 and 8 post immunization.

### HI Antibodies in Serum

All immunized chickens in each test group had significantly higher HI antibody titers (*p*<0.01) compared with chickens immunized with blank nanoparticles or with physiological saline (Table 6). At the same vaccine dosage, the HI antibody titers of chickens immunized with the LaSota live vaccine or the inactivated NDV vaccine were higher than those of the other groups in the first 4 weeks and then decreased, peaking at week 3 post immunization. However, the HI antibody titers of the chickens immunized orally or intranasally with NDV-CS-NPs increased continuously and peaked in week 5, during which the HI titers were significantly higher than those of the chickens immunized with the LaSota live vaccine or the inactivated NDV vaccine. Chickens immunized orally with NDV-CS-NPs had higher HI antibody titers than the intranasally immunized chickens. The HI antibody titers of the chickens immunized with LaSota live vaccine or the inactivated NDV vaccine decreased significantly at week 6 post immunization.

**Table 6:** HI antibody titers in sera of immunized SPF chickens (log_2_).

Groups	Weeks post immunization*							
	1	2	3	4	5	6	7	8
NDV-CS-NPs oral^A^	3.133±0.153	3.433±0.153	5.033±0.153	6.433±0.115	7.200±0.265	5.867±0.058	5.433±0.153	4.500±0.200
NDV-CS-NPs intranasal^AB^	4.867±0.153	5.000±0.100	5.867±0.153	6.300±0.100	7.067±0.153	5.233±0.153	4.767±0.115	3.267±0.058
LaSota live vaccine intranasal^C^	4.967±0.058	6.033±0.208	6.900±0.100	6.667±0.153	6.533±0.115	4.133±0.153	2.067±0.115	1.800±0.000
Inactivated NDV vaccine intramuscular^CD^	4.867±0.058	5.533±0.379	6.647±0.058	6.567±0.153	6.033±0.115	3.900±0.100	2.367±0.153	1.700±0.173
Blank CS-NPs control oral^E^	1.600±0.173	1.433±0.115	1.767±0.252	1.733±0.208	1.800±0.200	1.733±0.208	1.733±0.208	1.633±0.153
Physiological saline control^EF^	1.267±0.252	1.733±0.115	1.600±0.173	1.900±0.100	1.867±0.115	1.800±0.265	1.767±0.252	1.533±0.058

* IgG titers are presented as mean ± SD of five experiments at each time point in each group. Data with the different upper case superscript letters show extremely significant difference *(p*<0.01).

### IgA Antibody in Intestinal Mucus

**Figure 4:**
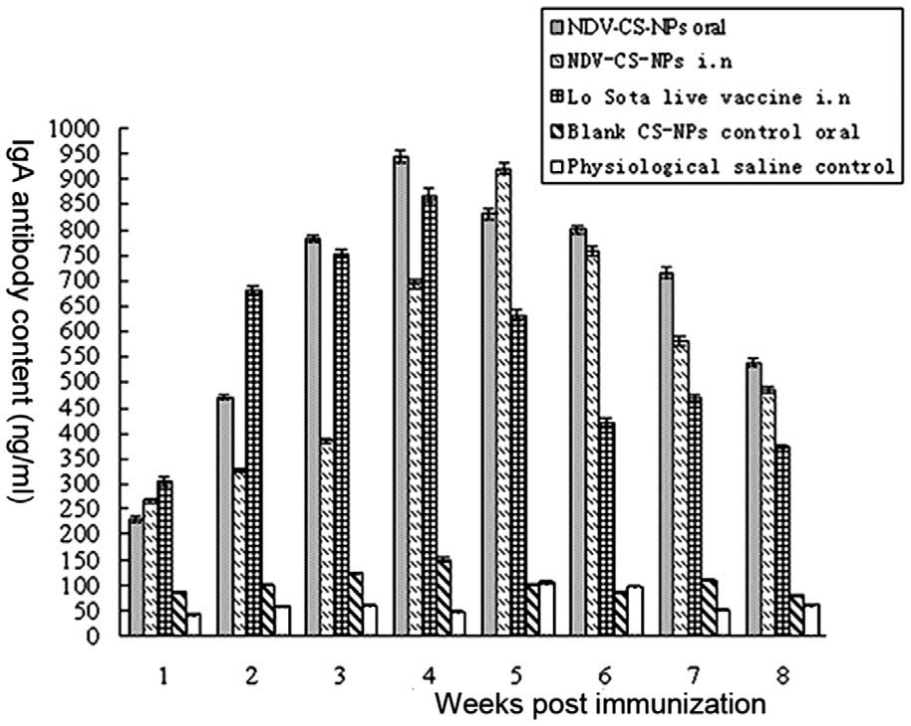
IgA antibody content in intestinal mucus of SPF chickens immunized with either NDV-CS-NPs orally, NDV-CS-NPs intranasally, LaSota live vaccine intranasally, blank CS-NPs control or physiological saline by oral route.

As shown in Figure 4, immunized chickens in each test group had significantly higher IgA antibody concentrations (*p*<0.01) compared with those immunized with blank chitosan nanoparticles or with physiological saline. The IgA antibody concentrations of chickens immunized intranasally with the LaSota live vaccine or orally with NDV-CS-NPs increased quickly and peaked at week 4 post immunization. After four weeks, chickens immunized with the LaSota live vaccine had a sharp decline in IgA antibody concentrations compared with those in the NDV-CS-NPs group. The IgA antibody concentrations of chickens immunized intranasally with NDV-CS-NPs increased slowly during the initial stage, peaked at week 5, and then decreased slowly. These findings indicated that the NDV-CS-NPs induced better a mucosal immune response compared with the LaSota live vaccine.

### Protective Efficacy of NDV-CS-NPs

Neither clinical signs nor mortality were observed in chickens immunized with NDV-CS-NPs after challenge with the highly virulent NDV strain F48E9. Feeding and drinking water were normal. Pathoanatomical results showed that there was no hemorrhage in the trachea and cloaca and only a tiny amount of hemorrhage in the lymphoid follicles of the duodenum and cecal tonsil. One of the chickens immunized with the LaSota live vaccine was dead and some had hemorrhages in their intestinal tract and proventriculus. Three of the chickens immunized with the inactivated NDV vaccine were dead and pathoanatomical results showed that there were severe hemorrhages in their intestinal tract and proventriculus. Chickens immunized with blank CS-NPs or with physiological saline were dead in 2–5 days after the challenge, and dead chickens with perse cockscombs demonstrated the typical pathological changes related to ND such as mucosal hemorrhages in the trachea, larynx, proventriculus, duodenum and the whole intestines.

## Discussion

The mucosal immune system of fowls includes the respiratory and alimentary tracts. Mucosal immunization induces mucosal immune response with S-IgA antibody and systemic humoral and cellular immune responses, whereas parenterally administered vaccines primarily stimulate systemic responses [27]–[29]. When compared with other immunization routes, mucosal delivery has many advantages, including a large epithelial surface with the presence of numerous microvilli, a porous endothelial membrane, highly vascularized mucosa facilitating absorption and ready accessibility [23], [30]. To enhance mucosal immune responses against NDV, we developed a novel mucosal delivery system for live virus vaccines based on chitosan nanoparticles (NDV-CS-NPs) in this study. NDV-CS-NPs vaccine provided better immune responses and better protection compared to the live and inactivated NDV vaccines.

Chitosan nanoparticles are an effective vehicle for the delivery of drugs in nasal administration [31]–[33]. It is believed that the positive charge on chitosan generated under physiological conditions is responsible for its enhanced bio-adhesivity and site-specific applications in controlled delivery systems [34], [35]. The ionic cross linking method was used to prepare NDV encapsulated in chitosan nanoparticles in this study because NDV is very sensitive to organic solvent and temperature. Western blotting analysis indicated that after encapsulation four NDV structural proteins (HN, F, P, and M) were still detected in NDV-CS-NPs, indicating that the ionic cross linking process of preparing NDV-CS-NPs had no significant effect on NDV proteins.

During the preparation of the NDV-CS-NPs, they were separated from the solution by centrifugation. The NDV-CS-NPs were freeze dried for zeta potential analysis. The zeta potential value is related to the stability behavior of the colloid. Generally speaking, colloids with a high zeta potential (negative or positive) are electrically stable whereas colloids with a low zeta potential tend to coagulate or flocculate. The colloids with a zeta potential of 40 to 60 mV are believed to be stable [36]. The measured zeta potential in this study was +2.84 mV. It should be pointed out that the vaccine used to immunize SPF chickens in this study contained 5% sucrose and skim milk, which prevented coagulation or flocculation of the NDV-CS-NPs.

The safety of the NDV-CS-NPs was tested by *in vitro* cytotoxicological analysis and safety test in chickens before animal immunizations. The survival rate was 91.5±5.3% and no significant changes in cell morphology were observed in comparison to the control cells, showing a low cytotoxicity of NDV-CS-NPs. In the safety test in chickens, 0.25 ml of NDV-CS-NPs was inoculated, and the dosage was four times the normal dosage. Feeding and drinking were normal and there were no nervous signs in inoculated chickens, showing a high level of safety of the NDV-CS-NPs in chickens.

Both humoral and cell-mediated immune responses play important roles in protecting chickens against NDV infection [37]–[40]. Antibodies directed against NDV usually appear 4–6 days post vaccination with live attenuated vaccines [41], which is supported by the HI antibody titers at week 1 in this study. Although high levels of systemic antibodies are associated with protection against ND [42], [43], haemagglutination inhibition (HI) antibody titers in serum are not directly correlated with the protection of chickens to an experimental NDV challenge [39], [44], [45]. This may explain why NDV-CS-NPs induced lower HI titers between weeks 1–4 post immunization compared to the LaSota live vaccine and the inactivated NDV vaccine, but conferred full protection of immunized chickens against the challenge with a virulent NDV strain in this study. These results may indicate the important roles of cell-mediated and mucosal immune responses in protecting chickens vaccinated against NDV [37], [38], [40], [41].

Cell-mediated immune response can be detected as early as 2–3 days post vaccination with NDV vaccines [40], [46]. Cell mediated immunity may be essential for virus clearance [47] even though the specific cell-mediated immunity is not sufficient by itself to protect against virulent NDV [40]. The results of the lymphocyte proliferation test in this study showed that NDV-CS-NPs induced the highest stimulation indices of T lymphocyte proliferation in immunized chickens at week 4 and also conferred full protection against the virulent NDV challenge. However, 3 out 5 chickens with the second highest stimulation indices of T lymphocyte proliferation post immunization with the inactivated NDV vaccine were dead whereas 1 out of 5 chickens with the third highest stimulation indices of T lymphocyte proliferation post immunization with the live NDV vaccine was dead, supporting the finding that the cell-mediated immune response is not sufficient by itself to protect against virulent NDV [40]. In addition, the local mucosal immune response plays an important role in developing protection for chickens vaccinated against NDV by inhibiting virus replication at the portal of entry for the virus [46], [48]. The intestinal IgA antibody concentrations in this study increased quickly in all test groups from week 1 to weeks 4 and 5 and then decreased, but this did not correlate with the protection of the vaccines, which supports the previous findings that IgM (not IgG or IgA) may be the class of antibody that is most actively involved in the clearance of NDV infection [49], [50].

Determining an optimum immunization dose is important in the vaccine efficacy trials for the efficient induction of immune responses in animals. Similar to the previous studies using chitosan nanoencapsulated vaccines [10], [51]–[53], one dose of the chitosan nanoencapsulated live NDV vaccine containing 10^6.5^ EID/0.1 ml was chosen in the vaccine efficacy trial in this study, and better immune responses were induced in chickens immunized with the chitosan nanoencapsulated live NDV vaccine compared to chickens immunized with the normal live NDV vaccine and the inactivated NDV vaccine. This immunization dose was greater than the minimum titer of 10^5.5^ EID50 for the NDV live vaccine as required by OIE [54]. The inoculation route for the challenge post immunization is another important factor in the live virus vaccine evaluation. As per the OIE procedure [54], vaccinated birds and control birds are challenged intramuscularly. Therefore, the NDV challenge was evaluated intramuscularly in this study. However, the mucosal routes may also be used to evaluate the NDV challenge [55]–[58].

In conclusion, NDV-CS-NPs have the advantages of live vaccines and inactivated vaccines, while compensating for their defects. The induction of significant mucosal immune responses after intranasal or oral administration of NDV-CS-NPs in chickens showed a range of potential applications of NDV-CS-NPs. More studies need to be conducted to further optimize these chitosan nanoparticles for use in commercial applications.
